# Notificación de defectos congénitos por brote del virus del Zika en Colombia, 2015-2017

**DOI:** 10.26633/RPSP.2019.38

**Published:** 2019-05-03

**Authors:** Fredy Orlando Mendivelso Duarte, Adriana Robayo García, Milena Rodríguez Bedoya, Gloria Suárez Rángel

**Affiliations:** 1 Centro de Medicina Basada en la Evidencia Keralty Centro de Medicina Basada en la Evidencia Keralty Bogotá Colombia Centro de Medicina Basada en la Evidencia Keralty, Bogotá, Colombia.; 2 Programa de Entrenamiento en Epidemiología de Campo (FETP) del Instituto Nacional de Salud de Colombia Programa de Entrenamiento en Epidemiología de Campo (FETP) del Instituto Nacional de Salud de Colombia Colombia Colombia Programa de Entrenamiento en Epidemiología de Campo (FETP) del Instituto Nacional de Salud de Colombia, Colombia.; 3 Fundación Universitaria Sanitas Fundación Universitaria Sanitas Bogotá Colombia Fundación Universitaria Sanitas, Bogotá, Colombia.

**Keywords:** Virus zika, anomalías congénitas, vigilancia sanitaria, salud pública, Colombia, Zika virus, congenital abnormalities, health surveillance, public health, Colombia, Zika virus, anormalidades congênitas, vigilância sanitária, saúde pública, Colombia

## Abstract

**Objetivo.:**

El brote por virus del Zika afectó a varios países tropicales durante 2015 y 2016. Esto obligó a crear estrategias de vigilancia intensificada de microcefalia y otros síndromes neurológicos. Se evaluó el efecto del brote por virus del Zika en la notificación de defectos congénitos en Colombia desde la perspectiva del sistema nacional de vigilancia.

**Métodos.:**

Se analizó la notificación nacional de recién nacidos con diferentes defectos congénitos y se determinaron las variaciones en la notificación atribuidas a la epidemia mediante un modelo semiparamétrico denominado “diferencia en diferencias” (DID).

**Resultados.:**

Un total de 18 234 casos por defectos congénitos fueron notificados en Colombia durante el período de estudio. La mayoría eran malformaciones congénitas (91,9%). El 82,3% se confirmó por diagnóstico clínico o nexo epidemiológico. En el caso de la microcefalia, se notificaron ocho casos nuevos por semana epidemiológica (coeficiente de notificación de casos [D] = 8,8; *P* = 0,000) y 32 casos por otras malformaciones congénitas anatómicas (D = 32,0; *P* = 0,000). El valor absoluto del estimador de diferencia en diferencias atribuido al brote por virus del Zika incrementó la notificación semanal de casos de microcefalia (DID = |-5,0|; *P* = 0,008) y malformaciones congénitas (DID = |-12,0|; *P* = 0,111).

**Conclusiones.:**

El brote por virus del Zika incrementó la notificación de recién nacidos con microcefalia, pero sin ninguna variación significativa en la notificación de otras malformaciones y defectos congénitos funcionales de origen sensorial o metabólico en el sistema de vigilancia.

## Abstract (en)

**Objective.:**

The Zika outbreak affected several tropical countries in 2015 and 2016, requiring the creation of intensified surveillance strategies for microcephaly and other neurological syndromes. The effect of the Zika outbreak on the reporting of birth defects in Colombia was evaluated from the perspective of the national surveillance system.

**Methods.:**

National reporting of newborns with different birth defects was analyzed; variations in reporting attributed to the epidemic were determined through difference in differences (DID), a semiparametric model.

**Results.:**

During the period of study, 18,234 cases of birth defects were reported in Colombia. The majority were congenital malformations (91.9%), and 82.3% was confirmed by clinical diagnosis or epidemiological link. In the case of microcephaly, eight new cases per epidemiological week were reported (coefficient of case reporting [D] = 8.8; *P* = 0.000) and 32 cases from other congenital anatomical malformations (D = 32.0; *P* = 0.000). The absolute value of the difference in differences estimator attributed to the Zika outbreak increased weekly case reporting of microcephaly (DID = |-5.0|; *P* = 0.008) and congenital malformations (DID = |-12.0|; *P* = 0.111).

**Conclusions.:**

The Zika outbreak increased reporting of newborns with microcephaly, but caused no significant variation in reporting of other malformations and functional birth defects of sensory or metabolic origin in the surveillance system.

## RESUMO

**Objetivo.:**

O surto de vírus zika afetou vários países tropicais de 2015 a 2016. Fez-se necessário elaborar estratégias de vigilância intensificada da microcefalia e outras síndromes neurológicas. O presente estudo avaliou o impacto do surto de vírus zika na notificação de defeitos congênitos no sistema nacional de vigilância da Colômbia.

**Métodos.:**

A notificação nacional de recém-nascidos com defeitos congênitos foi analisada e a variação na notificação atribuída à epidemia de zika foi determinada com o uso de um modelo semiparamétrico de diferença em diferenças (DD).

**Resultados.:**

Ao todo, 18.234 casos de defeitos congênitos foram notificados na Colômbia no período considerado. Os casos, na sua maioria, foram de malformações congênitas (91,9%), sendo 82,3% confirmados por diagnóstico clínico ou nexo epidemiológico. Foram notificados oito casos novos de microcefalia por semana epidemiológica (coeficiente de notificação de casos [D] = 8,8; *P* = 0,000) e 32 casos de outras malformações congênitas anatômicas (D = 32,0; *P* = 0,000). O valor absoluto do estimador do método de diferença em diferenças atribuído ao surto de vírus zika indicou um aumento na notificação semanal de casos de microcefalia (DD = |–5,0|; *P* = 0,008) e malformações congênitas (DD = |–12,0|; *P* = 0,111).

**Conclusões.:**

O surto de vírus zika ocasionou um aumento na notificação de recém-nascidos com microcefalia, mas não houve variação significativa na notificação de outras malformações e defeitos congênitos funcionais de origem sensorial ou metabólica no sistema de vigilância.

Los defectos congénitos (DC) son causa importante de mortalidad infantil, enfermedad crónica y discapacidad en todo el mundo (1). En 2010, la Asamblea Mundial de la Salud adoptó la resolución sobre defectos de nacimiento en la que pidió fomentar la prevención primaria y la salud de los niños con anomalías congénitas mediante implementación de diferentes estrategias como el fortalecimiento de sistemas de registro, vigilancia, investigación en etiología y rehabilitación (2). Las causas de estos defectos son múltiples y de causas clínicas, genéticas e infecciosas. A nivel mundial, nacen 7,6 millones de niños cada año con malformaciones genéticas o congénitas graves; 90% en países de ingresos medios o bajos. Pese a estas cifras, y aunque muchas malformaciones son evidentes, es difícil reunir datos precisos sobre la prevalencia, dado que existe una gran diversidad de malformaciones y en muchos casos no llegan a diagnosticarse de forma clara (3). La Organización Mundial de la Salud (OMS) estima que 276 000 recién nacidos (RN) mueren cada año debido a anomalías congénitas y los niños que logran superar esta etapa sufren diferentes discapacidades y afectación en su calidad de vida con impacto en su núcleo familiar, instituciones, sistemas de salud y la sociedad. Son pocas las alternativas con efectividad comprobada para la prevención de anomalías congénitas. Entre ellas están la vacunación, la ingesta de ácido fólico y el enriquecimiento de alimentos básicos o el suministro de complementos, así como los cuidados prenatales adecuados (4).

En 2015, el mundo se vio afectado por un brote de infección por virus del Zika (ZIKV, por sus siglas en inglés), que comenzó en las islas del Pacífico sur y se propagó a Brasil y otros países de las zonas tropicales de América Latina (5). Aunque el comportamiento clínico de la enfermedad es el de un síndrome febril agudo, la mayor preocupación fue la confirmación de su efecto neurotrópico y la relación causal directa con microcefalia en RN de las zonas con una mayor incidencia por el brote en Brasil (6, 7). Se están realizando múltiples estudios clínicos en ciencias básicas y otras áreas para generar más evidencia. Esto no solo ayudará a entender los mecanismos de afectación neurológica, sino la aparición de otros trastornos neurológicos relacionados (8-10).

El efecto que tuvo el brote por ZIKV en los procesos de notificación y la respuesta de los sistemas de vigilancia de DC han sido poco estudiados (11). En Colombia, antes de la aparición del brote, se realizaba la notificación individual de DC al Sistema Nacional de Vigilancia (Sivigila); en ese entonces, se observaban diferentes patrones de comportamiento entre zonas geográficas. La aparición del brote ZIKV suponía un incremento exponencial en la notificación de casos de DC concentrado en zonas geográficas más desarrolladas y con la tecnología suficiente para sospechar o confirmar casos (12). El análisis de brotes o emergencias en salud pública para enfermedades crónicas no trasmisibles (ENT) es infrecuente y presenta variaciones metodológicas radicales si se compara con el estudio de situación de brotes en enfermedades infecciosas. Entre otras razones, esto ocurre porque los desenlaces en salud y el efecto de intervenciones para su control presentan una dinámica diferente y de resultados solo observable en el muy largo plazo (13).

El objetivo del estudio fue evaluar el efecto o variación inducida por el brote ZIKV en el comportamiento de la notificación de RN con defectos congénitos (malformaciones anatómicas incluida la microcefalia y DC funcionales de origen sensorial o metabólicos) registrados en el sistema nacional de vigilancia de Colombia.

## MATERIALES Y MÉTODOS

Se realizó un estudio ecológico con aplicación de un modelo semiparamétrico para estimar la magnitud del efecto y variación en la notificación de casos de RN con DC durante el brote por ZIKV. Se vincularon la totalidad de casos de DC y ZIKV en gestantes notificados al Sivigila durante 2013-2017 para la generación del modelo. Se incluyeron variables demográficas, clínicas y medioambientales en el análisis. Los DC se clasificaron según la Clasificación Internacional de Enfermedades, décima edición (CIE-10) como defectos funcionales metabólicos (E000 a E889), defectos funcionales sensoriales (H900 a H355) y malformaciones congénitas (Q000 a Q960). La declaración oficial de brote por ZIKV en Colombia ocurrió en la semana epidemiológica (SE) 40 de 2015 y el pico de notificación de casos se presentó en la SE 4 del 2016.

### Análisis estadístico

Las variables cuantitativas se analizaron mediante medidas de frecuencia, tendencia central y dispersión; los datos categóricos, mediante proporciones. Los supuestos de distribución normal se analizaron con el test no paramétrico de Shapiro-Wilk; el análisis bivariado, con el estadístico *X*2 y prueba exacta de Fisher. Según la distribución de variables continuas, se calcularon pruebas estadísticas de t-student o U de Mann Whitney. En el contraste de hipótesis se consideró estadísticamente significativo un valor de *P* <0,05. Para cada estimador se calcularon intervalos de confianza del 95% (IC95%).

### Modelo de diferencia en diferencias

Cuando ocurre un evento fortuito, como por ejemplo la aparición del brote ZIKV, podemos hablar de las condiciones necesarias para plantear un experimento natural que genera una asignación aleatoria de la exposición poblacional (en este caso, la probabilidad de ser infectado por ZIKV durante la fase epidémica). En estas condiciones, el análisis de diferencia en diferencias (DID) mide el cambio inducido por ZIKV en la notificación de DC en función del tiempo (fase epidémica) al comparar las tendencias antes y después del brote. El estimador DID cuantifica esta diferencia entre infectados, ZIKV (+), y no infectados, ZIKV (-).

El modelo DID se define para efectos prácticos como sigue:

$$\begin{array}{rr} \text{ZIKV(+)} & \text{ZIKV(-)}\\ t\;=\;1\;Y1|D\;=\;1 & Y1|D\;=\;0\\ t\;=\;2\;Y2|D\;=\;1 & Y2|D\;=\;0 \end{array}$$

Donde *t = 1* es el período anterior a la aparición del brote ZIKV (*D*) y *t = 2* es el período posterior al brote. El modelo DID calcula el estimador de diferencias así:

τdif en dif= [E(Y2|D=1)−E(Y1|D=1)]−[E(Y2|D=0)−E(Y1|D=0)]

El primer factor de la ecuación estima el cambio esperado en la notificación de DC entre el período anterior y el posterior a la aparición del brote en el grupo ZIKV(+) y el segundo factor estima el cambio esperado en la notificación de DC entre el período anterior y el posterior al brote en el grupo ZIKV(-). Dicho de otra forma:

$${\hat{\tau }}_{dif\;en\;dif}=\;\left(\Delta \hat{Y}|D=1)-(\Delta \hat{Y}|D=0\right)$$

Al reescribir la ecuación:

$${\hat{\tau }}_{dif\;en\;dif}=\;\left[\left({\hat{Y}}_{2}\text{|}D=1\right)-\left({\hat{Y}}_{2}\text{|}D=0\right)\;\right]-\left[\left({\hat{Y}}_{1}\text{|}D=1\right)-\;\left({\hat{Y}}_{1}\text{|}D=0\right)\right]$$

El primer factor estima la diferencia promedio entre el grupo ZIKV(+) y ZIKV(-) en el período de observación *t = 2* y el segundo factor estima la diferencia promedio entre el grupo ZIKV(+) y ZIKV(-) en el período de observación *t = 1*.

Para cada subgrupo de notificación de DC (microcefalia, malformaciones congénitas, defectos funcionales metabólicos y sensoriales) se calculó un modelo DID derivado de la siguiente forma:

$$Y_{i}=\;\beta_{0}+\;\beta_{1}X_{1}+\;\beta_{2}X_{2}+\;\beta_{3}X_{1}X_{2}+\;\delta$$

Donde:

*Y*_i_: número de casos notificados del subgrupo de DC por SE*.*

*X*_1_: período de observación (t_(1|0)_), antes o después de la aparición del brote.

*X*_2_: *S*E cuando aparece del brote (D_(1|0)_)*.*

*X*_1_*X*_2_: interacción entre la notificación promedio de casos antes y después del brote.

*δ*: error aleatorio.

$\hat{\beta_{0}}$: número promedio de casos notificados en el período anterior al brote.

$\hat{\beta_{0}}+\;\hat{\beta_{1}}$: es el resultado promedio de casos notificados para el grupo anterior al brote durante la fase epidémica.

$\hat{\beta_{2}}$: es el efecto estimado del brote sobre la notificación del evento. Mide la diferencia en el número de casos notificados entre los grupos ZIKV(+) y ZIKV(-). Se denomina “d” dentro de le representación gráfica de coeficientes del modelo.

$\hat{\beta_{0}}+\;\hat{\beta_{2}}$: es el resultado promedio de casos notificados para el grupo posterior al brote en la línea de base.

$\hat{\beta_{0}}+\;\hat{\beta_{1}}+\;\hat{\beta_{2}}+\;\hat{\beta_{3}}$: es el resultado promedio de casos notificados para el grupo tratado en la fase epidémica.

$\hat{\beta_{3}}$: su valor absoluto es el estimador DID de impacto.

### Aspectos éticos

El protocolo de investigación fue evaluado y aprobado para su ejecución por el Comité de Ética Científico del Instituto Nacional de Salud de Colombia quienes suministraron la información necesaria para su elaboración.

## RESULTADOS

Un total de 18 234 casos con DC fueron notificados al Sivigila durante el período de análisis (SE 1 de 2015 a SE 36 de 2017). La mayoría eran de sexo masculino (52,8%), con una edad promedio de 1,2 meses, residencia en zonas urbanas (78,4%) y aseguramiento público (54,8%). El 91,9% de los casos notificados fueron malformaciones congénitas y la mayoría se confirmó por diagnóstico clínico (82,3%). El 71,0% procedían de ciudades ubicadas a ≤ 2 200 metros sobre el nivel del mar (msnm) y, según estudios de entomología en Colombia, no es posible encontrar el vector para ZIKV y otras arbovirosis en alturas superiores (cuadro 1). Las madres de estos RN eran jóvenes (con una media de edad de 26 años), de las cuales 10,0% eran niñas y adolescentes (10 a 17 años). En antecedentes de fecundidad, el número de hijos fue uno en 46,9% de los casos, sin aborto previo en 81,1% y sin antecedente de mortalidad perinatal en 90,4% (cuadro 2).

**FIGURA 1:**
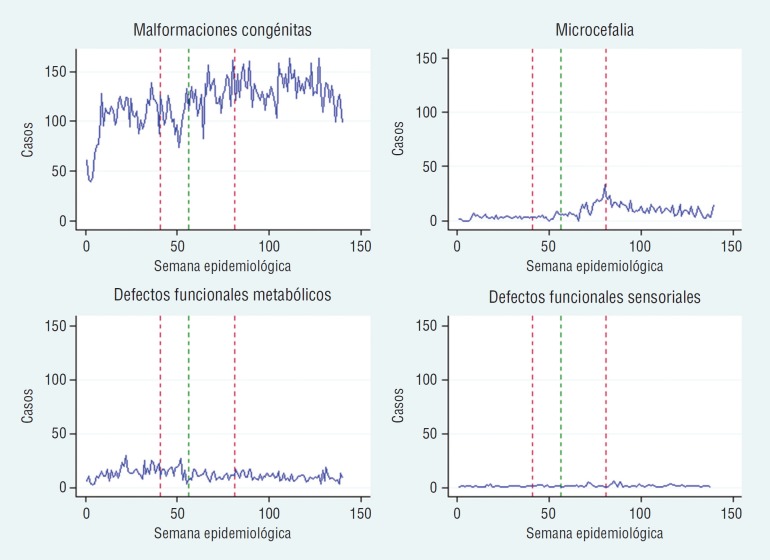
Tendencia en la notificación de defectos congénitos en Colombia

Los datos recolectados por Sivigila durante aproximadamente 140 SE mostraron un incremento en la notificación de casos de malformaciones congénitas, pero no para trastornos funcionales metabólicos o sensitivos. Los gráficos de tendencia en la notificación de DC revelaron un aumento que coincidió con el pico de notificación por ZIKV. Lo llamativo de esta tendencia fue que se mantuvo durante gran parte de la fase posepidémica (figura 1).

**CUADRO 1:** Características demográficas de los casos notificados por defectos congénitos

Características		Valor	
		n	%
Sexo			
	Masculino	9628	52,8
	Femenino	8282	45,4
	Indeterminado	324	1,8
Área de procedencia de la madre			
	Urbana	14288	78,4
	Rural	3946	21,6
Afiliación en salud			
	Subsidiada	9996	54,8
	Contributiva	8238	45,2
Diagnóstico			
	Malformaciones congénitas	16765	91,9
	Defectos funcionales metabólicos	1686	9,2
	Microcefalia	1050	5,8
	Defectos funcionales sensitivos	174	0,9
Tipo de caso			
	Confirmado por clínica	16105	88,3
	Confirmado por laboratorio	1402	7,7
	Probable	727	4,0
Paciente hospitalizado			
	Sí	12370	67,8
	No	5864	32,2
Condición final			
	Vivo	15332	84,1
	Muerto	2800	15,4
	Sin dato	102	0,5
Altura del municipio de procedencia		12915	71,0
	≤ 2200 msnm	5267	29,0
	≥ 2200 msnm		
		Media	DE
Edad al realizar la notificación (en meses)		1,2	2,1
Edad gestacional al nacer (en semanas)		36,3	4,7
Peso al nacer (en gramos)		2656,3	848,5

msnm, metros sobre el nivel del mar; DE, desviaciones estándares.

**CUADRO 2:** Características demográficas, antecedentes obstétricos y lugar de procedencia de las madres de los casos notificados por defectos congénitos

Características		Valor	
		n	%
Número de embarazos			
	Uno	7844	43,0
	Dos	5174	28,4
	Tres	5261	28,6
Número de hijos nacidos vivos			
	Ninguno	1229	6,8
	Uno	8548	46,9
	Dos	5016	27,5
	Tres o más	3424	18,8
Antecedente de aborto espontáneo			
	Ninguno	14788	81,1
	Uno	2705	14,8
	Dos	579	3,2
	Tres o más	162	0,9
Antecedente de mortalidad perinatal			
	Ninguno	16488	90,4
	Uno	1563	8,6
	Dos	156	0,9
	Tres o más	27	0,2
Interrupción voluntaria del embarazo (IVE)			
	No	17270	94,7
	Sí	964	5,3
Antecedente de enfermedades crónicas			
	No	17868	80,0
	Sí	366	2,0
Antecedente de embarazo múltiple			
	No	17621	96,6
	Sí	613	3,4
Consumo o ingesta durante el embarazo			
	Ácido fólico	13211	72,4
	Bebidas alcohólicas	462	2,5
	Cigarrillo	208	1,1
	Sustancias psicoactivas	129	0,8
	Exposición a agentes teratógenos	364	2,0
Toma de muestra para tamizaje			
	TORCH	3120	17,1
	Anticuerpos IgM para rubéola	185	1,2
	Anticuerpos IgM para toxoplasmosis	245	1,5
	Anticuerpos IgM para citomegalovirus	149	0,9
	Anticuerpos IgM para herpes	112	0,7
	TSH de cordón	8298	45,5
	TSH de talón	485	2,7
		Media	DE
Edad de la madre (años)		26,10	7,3
Edad gestacional de la IVE (semanas)		24,38	7,4

TORCH, toxoplasmosis, rubéola, citomegalovirus, herpes; THS; tirotropina; DE, desviaciones estándares; IVE, interrupción voluntaria del embarazo.

La notificación de microcefalia también mostró el mismo incremento, sobre todo a partir del pico de notificación de ZIKV (SE 4 de 2016) y se mantuvo elevada hasta muy cerca de la declaración de cierre de la situación de brote en Colombia (figura 1).

**FIGURA 2:**
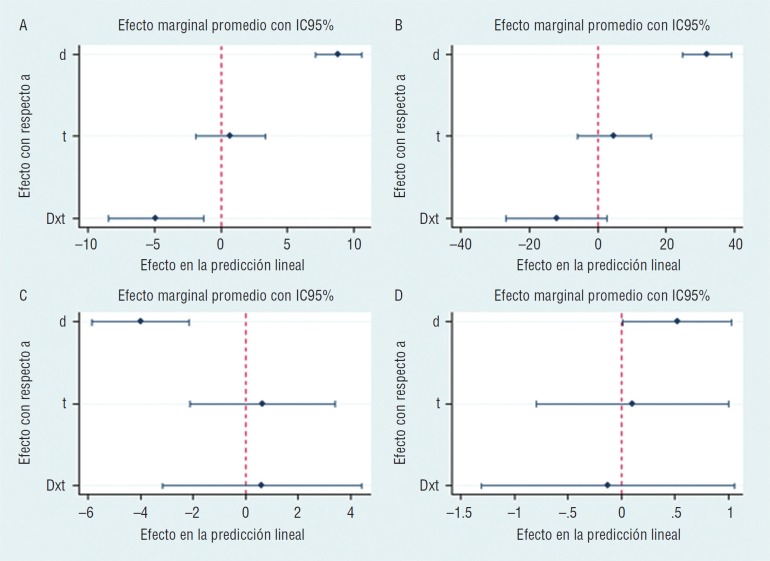
Coeficientes de estimación del efecto del brote por ZIKV (d), período de observación (t) e interacción en las fases preaparición y posaparición del brote (Dxt) en la notificación de casos semanales de defectos congénitos al sistema nacional de vigilancia de Colombia. **A,** notificación de casos de microcefalia; **B,** notificación de casos de malformaciones congénitas; **C,** notificación de casos de trastornos funcionales metabólicos; **D,** notificación de casos de trastornos funcionales sensoriales

El primer modelo DID evaluó la predicción lineal de notificación de casos de microcefalia. En este modelo, el coeficiente asociado al efecto del brote sobre la notificación de microcefalia (D = 8,8; *P* = 0,000) indicó la presencia de nueve casos más de microcefalia por SE durante la fase epidémica (aproximadamente 360 casos notificados al sistema durante las 40 semanas en que el país se mantuvo en situación de brote) (figura 2).

$$Y_{microcefalia}=\;2,6+\;0,71X_{1}+\;8,8X_{2}+\left(-4,8\right)X_{1}X_{2}+\;\delta$$

Dado que durante un brote existe una fase creciente de notificación, un pico y un descenso posterior de duración variable, no se debería limitar el análisis al efecto inicial (fase creciente) porque tiende a sobreestimar el efecto real del brote. Para resolver esta brecha, se calculó el estimador (D = |-4,9|; *P* = 0,008) el cual considera para su cálculo los efectos residuales de los casos notificados por microcefalia en la totalidad de la curva epidémica. En total, se estimó que el brote causó un incremento de cinco casos notificados por SE para microcefalia durante toda la fase epidémica en el país o, dicho de otra forma, el incremento neto en la notificación fue de aproximadamente 200 casos más de microcefalia notificados al sistema por efecto del brote de ZIKV (figura 2).

La derivación del segundo modelo para la notificación de malformaciones congénitas también mostró un incremento positivo en la estimación de casos notificados al Sivigila por efecto del brote de ZIKV (D = 31,95; *P* = 0,000), pero al calcular el estimador de efecto neto (DID = |-11,95|; *P *= 0,111) resultó menor y estadísticamente no significativo, lo que se pudo entender como la consecuencia de una mayor notificación de casos durante la primera fase del brote:

$$Y_{malformaciones\;congénitas}=\;100,8+\;4,7X_{1}+\;31,9X_{2}+\left(-11,9\right)X_{1}X_{2}+\;\delta$$

Los modelos restantes para notificación de defectos funcionales metabólicos y sensoriales revelaron que el efecto neto del brote de ZIKV no tuvo impacto al modificar o alterar el número de casos notificados por semana en ambos subgrupos durante la epidemia:

$$\begin{array}{l} Y_{D.funcionales\;metabólicos}=\;14,2+\;0,6X_{1}+\left(-4,0\right)X_{2}+0,62X_{1}X_{2}+\;\delta \\ Y_{D.funcionales\;sensoriales}=\;1,6+\;2,1X_{1}+1,6X_{2}+(-0,12)X_{1}X_{2}+\;\delta \end{array}$$

## DISCUSIÓN

La vigilancia en salud pública es un proceso que requiere el esfuerzo y la colaboración de muchos actores del sistema de salud (14). La importancia de esta actividad se centra en la posibilidad de monitorizar y responder en muchos casos a brotes y emergencias con impactos variados sobre la población general y los sistemas de salud (15-17). Uno de los principales retos para los países afectados por el brote fue la necesidad de desarrollar, validar y difundir con celeridad un protocolo para la vigilancia de casos de infección por el zika y otros para la vigilancia de síndrome neurológicos en población general, con énfasis en la microcefalia y el síndrome de Guillain-Barre (18). En la práctica, se trataba de crear una estrategia para vigilancia de arbovirosis como sucede, por ejemplo, en brotes de fiebre por el virus del dengue, pero que requería la vigilancia y el análisis concurrentes de los efectos neurológicos deletéreos asociados a la infección por ZIKV por la asociación directa de DC en RN (19-22).

Los resultados mostraron un claro incremento en la notificación de DC, sobre todo en los casos de microcefalia relacionados con la presencia de ZIKV (8, 23). Si bien las autoridades nacionales de salud esperaban un mayor número de casos notificados al observado por el sistema de vigilancia, no fue posible dilucidar si estos casos notificados en realidad estuvieron originados por una infección ZIKV(+), dado el bajo porcentaje de confirmación por laboratorio (24). La metodología propuesta nos permitió incorporar en el análisis el efecto de toda la fase epidémica del brote del zika sobre las consecuencias en el corto y mediano plazo detectadas por el sistema de vigilancia de DC; se identificó un claro sesgo hacia la notificación de las microcefalias y por ahora, muy poca variación en otras afectaciones de interés como los trastornos funcionales metabólicos y sensoriales. Esto se debe tal vez a la baja posibilidad de confirmar el diagnóstico, pero con la clara sensación, según reportes de otros estudios que evalúan cohortes de gestantes con zika, que aún existe baja notificación de estos casos (25).

La fase epidémica por el virus del Zika comprendió la SE 41 de 2015 hasta la SE 30 de 2016 (franja roja). La línea de color verde corresponde al pico de notificación de casos por ZIKV.

Elaboración propia a partir de los resultados presentados.

Dentro de las limitaciones más importantes del estudio estuvo la imposibilidad de validar si los casos notificados por DC tenían una prueba ZIKV(+), lo cual nos limitó a clasificar los casos por “hallazgos clínicos al examen físico”. Esto, a su vez, aumentó la incertidumbre frente a los defectos funcionales a pesar de que se permiten ajustes en el Sivigila tras la primera notificación durante el seguimiento que realiza el equipo de vigilancia de la notificación reportada. Vale la pena recordar que, dentro de la codificación de malformaciones congénitas (Capítulo XVII del CIE-10, códigos Q000-Q990), existe gran cantidad de deformidades y anomalías cromosómicas que causan DC funcionales y que para su confirmación requieren estudios muy especializados, análisis de biomarcadores y estudios de genómica de alta complejidad muchos de los cuales no están disponibles al momento de la notificación causando al sesgo de detección orientado a defectos anatómicos visibles durante el examen físico del RN o lactante pequeño. Los datos complementarios en la notificación, sobre todo los que registran exposiciones de riesgo (consumo de sustancias o exposición a agentes teratógenos) presentaban bajo registro y limitaban la posibilidad de explorar otras asociaciones con la incidencia de DC diferentes al brote de ZIKV. Otro aspecto no evaluado en este estudio fue determinar si existió alguna relación en las cifras de aborto espontáneo antes de las 20 semanas de gestación durante la fase epidémica del brote, dado que estos casos no hacen parte de la notificación de RN.

## CONCLUSIONES

Se evidencia la relación positiva entre infección por ZIKV en gestantes y aparición de microcefalia durante el brote hasta cuarenta semanas posteriores al pico de notificación por ZIKV. No hubo variación en la notificación de defectos funcionales metabólicos y sensoriales. La baja proporción de confirmación mediante pruebas especializadas condicionan al sistema de vigilancia de defectos congénitos a recibir, en su mayoría, notificaciones por malformaciones estructurales y muy pocas por trastornos funcionales metabólicos o sensoriales. El proceso de notificación a un sistema de vigilancia requiere entrenamiento permanente en protocolos de vigilancia, validación y calidad del registro en los datos que ingresan al sistema para que las autoridades nacionales puedan realizar los análisis a profundidad. La posibilidad de implementar nuevas metodologías de análisis resulta una alternativa innovadora para mejorar la toma de decisiones en salud pública.

## Contribución de los autores.

Todos los autores (FM, AR, MR, GS) concibieron el estudio original, recolectaron consolidaron y analizaron los datos, interpretaron los resultados, escribieron y revisaron el manuscrito, atendieron la solicitud de ajustes realizadas por los evaluadores y todos los autores revisaron y aprobaron la versión final.

IC95%, intervalo de confianza de 95%.

Elaboración propia a partir de los resultados presentados.

## Agradecimientos.

Los autores agradecen a la Dirección General y a la Subdirección de Vigilancia y Análisis de Riesgo en Salud Pública del Instituto Nacional de Salud de Colombia por su colaboración y abnegada labor.

## Declaración.

Las opiniones expresadas en este manuscrito son responsabilidad de los autores y no reflejan necesariamente los criterios ni la política de la *RPSP/ PAJPH* y/o de la OPS.
